# Study protocol for a randomised controlled trial to determine the effectiveness of a mHealth application as a family supportive tool in paediatric otolaryngology perioperative process (TONAPP)

**DOI:** 10.1186/s13063-023-07376-z

**Published:** 2023-05-25

**Authors:** Raffaella Dobrina, Andrea Cassone, Margherita Dal Cin, Luca Ronfani, Manuela Giangreco, Silvana Schreiber, Sara Zanchiello, Anja Starec, Laura Brunelli, Liza Vecchi Brumatti, Livia Bicego

**Affiliations:** 1 Healthcare Professions Department, Institute for Maternal and Child Health IRCCS Burlo Garofolo, Trieste, Italy; 2 Department of Health Prevention, Azienda Sanitaria Universitaria Giuliano Isontina, Trieste, Italy; 3 Clinical Epidemiology and Public Health Research Unit, Institute for Maternal and Child Health IRCCS Burlo Garofolo, Trieste, Italy; 4 Area Science Park, Trieste, Italy; 5 Scientific Direction, Institute for Maternal and Child Health IRCCS Burlo Garofolo, Trieste, Italy; 6 University of Trieste, Trieste, Italy

**Keywords:** mHealth, Tonsillectomy, Education

## Abstract

**Background:**

Otorhinolaryngology (ORL) surgery is common in children, but hospitalisation, surgery, and home care after discharge are stressful experiences for young patients and their family caregivers. Findings from literature highlight a lack of time in hospitals to support ORL surgery children and their caregivers through the perioperative process, along with the risks of caregivers’ autonomous web or social media resources investigation. Therefore, this study aims to evaluate the effectiveness of a mobile health application with content to support ORL patients and their caregivers in the perioperative period to reduce caregiver anxiety and child distress compared to standard care.

**Methods:**

An open-label, two-arm randomised control trial design is being adopted. The intervention consists of a mobile health application with content to support ORL patients and their caregivers during the perioperative period. One hundred eighty participants will be enrolled and randomly assigned to the experimental group using the mHealth application or the control group. The control group receives standard information and education about the ORL perioperative period from healthcare providers orally or through brochures. The primary outcome is the difference between the intervention and control groups in preoperative caregiver state anxiety. Secondary outcome measures include children’s distress before surgery and family preparation for hospitalisation.

**Discussion:**

The results of this study will be critical to the implementation of a new and safe model for the management of care and education in paediatrics. This model can achieve positive organisational and health outcomes by supporting continuity of care and empowering citizens to have informed participation and satisfaction in paediatric health promotion and management.

**Trial registration:**

Trial identifier: NCT05460689 registry name: ClinicalTrials.gov. Date of registration: July 15, 2022. Last update posted: February 23, 2023.

## Administrative information

Note: the numbers in curly brackets in this protocol refer to SPIRIT checklist item numbers. The order of the items has been modified to group similar items (see http://www.equator-network.org/reporting-guidelines/spirit-2013-statement-defining-standard-protocol-items-for-clinical-trials/).

**Table:** 

Title {1}	Effectiveness of a mHealth Application as a Family Supportive Tool in Pediatric Otolaryngology Perioperative Process (TONAPP)".
Trial registration {2a and 2b}.	NCT05460689
Protocol version {3}	03/22 (March 2022) version 1
Funding {4}	The personalization of the device (mHealth app) in its contents and features, part of an information platform, was founded by ARGO system, a protocol agreement signed in 2018 among the Friuli Venezia Giulia Region, the Italian Ministry of Education University and Research, and the Italian Ministry of Economic Development. Moreover, this work was supported by the Italian Ministry of Health, through contributions given to the Institute for Maternal and Child Health IRCCS Burlo Garofolo, Trieste, Italy.
Author details {5a}	RD, AC, SS, LB: Healthcare professions department, Institute for maternal and child health IRCCS Burlo Garofolo, Trieste, Italy. MDC: Department of health prevention, Azienda Sanitaria Universitaria Giuliano Isontina; LR, MG: Clinical Epidemiology and Public Health Research Unit, Institute for maternal and child health IRCCS Burlo Garofolo, Trieste, Italy; SZ, AS: Area Science Park, Trieste, Italy; LB: University of Trieste, Trieste, Italy. LVB: Scientific Direction, Institute for maternal and child health IRCCS Burlo Garofolo, Trieste, Italy
Name and contact information for the trial sponsor {5b}	This work is supported by the Ministry of Health, Rome—Italy, in collaboration with the Institute for Maternal and Child Health IRCCS Burlo Garofolo, Trieste – Italy.
Role of sponsor {5c}	This study is supported by the Institute for Maternal and Child Health IRCCS Burlo Garofolo, Trieste, Italy, through the contribution from the Italian Ministry of Health (Ricerca Corrente 03/22). Caregivers of patients are enrolled at the Institute of Maternal and Child Health IRCCS Burlo Garofolo. The role of the sponsor is to ensure that adequate arrangements are made for the initiation and management of the study and that liability insurance and legal liability are in place. In addition, the project underlying the study is funded by the Area Science Park as part of the complex project "ARGO System", a protocol agreement signed in 2018 between the Friuli Venezia Giulia Region, the Italian Ministry of Education, University and Research and the Italian Ministry of Economic Development.

## Introduction

### Background and rationale {6a}

Tonsillectomy and/or adenoidectomy with or without insertion of a tympanostomy tube are common surgeries in children. However, authors report children and their family are distressed by these perioperative processes. Fear of the unknown may challenge the preoperative period, while pain and other possible complications such as fever, vomiting, limited oral intake, or bleeding may complicate the postoperative management at home [1, 2]. Moreover, parental anxiety has been found to worsen children’s perceptions of pain, perioperative distress, and recovery [3].

Preparing children and families for hospitalisation, surgery, and postoperative management at home has been shown to improve perioperative outcomes [2]. However, not all individuals understand and can benefit from the information provided by healthcarers. In fact, higher levels of anxiety in the perioperative process have been associated with individuals with low health literacy [4]. According to Sørensen and colleagues [5], “Health literacy is linked to literacy and entails people’s knowledge, motivation and competences to access, understand, appraise, and apply health information in order to make judgements and take decisions in everyday life concerning healthcare, disease prevention and health promotion to maintain or improve quality of life during the life course”. In paediatric surgery, this specifically includes the ability of parents or other caregivers to understand the rationale for surgery to treat their child’s condition, consent forms, preoperative and postoperative instructions, and/or drugs prescriptions. Low health literacy in paediatrics has also been found to affect health-related outcomes and child safety, as well as with preventable hospitalisations [6]. Therefore, authors advocate the health literacy benefits of a family-centred perioperative care and education. The information needs of children and their caregivers, their health literacy, learning and coping styles, and the family’s experiences in the hospital, such as with invasive or painful procedures, are all factors to consider in this process for each family unit [4, 7]. However, patient- and family-centred education and support is a complex and time-consuming care interventions, whereas some surgical procedures, such as tonsillectomy, are characterised by a short hospital stay, thus limiting the time that healthcare providers can devote to this program [8]. Moreover, unmet information needs may lead parents or other caregivers to expose themselves to health-related misinformation through autonomous web and common social media resources investigation [9, 10]. Therefore, health systems have tested different types of formats, content, and delivery of education to meet client needs, time availability, and effectiveness. A systematic review that aimed at exploring the types and benefits of patient/parent education programs prior to tonsillectomy identified several educational methods for tonsillectomy management, such as verbal information for caregivers, informational brochures, videos, Internet resources, mobile health applications (mHealth apps), and mobile reminder text messages. In particular, mHealth apps and text messaging were consistently shown to be beneficial to patients in terms of pain, stress and compliance, and the authors recommend their use in clinical settings where other types of perioperative education are lacking or do not lead to positive outcomes [2, 11].

In particular, mHealth apps are an essential element of electronic health and consist of medical information available through mobile phones or other wireless devices that can be used by patients or healthcarers. Their use is increasing and evolving in a variety of functions and positive outcomes related to the improving the well-being of individuals, including diagnostics and clinical decision making, healthy lifestyles interventions, disease management, and self-care [12]. However, findings from literature reviews addressing current research on the use of clinical apps indicate that further randomised controlled trials with larger sample size (> 60) and improved rigour in study design and methods are needed to confirm positive outcomes [13, 14]. Moreover, in a literature review of qualitative studies exploring patients’ perceptions of mHealth apps, results reported customer satisfaction and engagement in their own care through the use of apps. However, weaknesses of mHealth apps have also been reported, including lack of disease-specific, cultural, and health literacy level personalisation, accessibility, and evidence-based information [15]. Furthermore, to our knowledge, there are few studies testing mHealth apps for family-centred perioperative tonsillectomy and/or adenoidectomy with or without tympanostomy tube insertion.

Therefore, this study aims to investigate the effectiveness of an mHealth app in supporting caregivers of children undergoing tonsillectomy and/or adenoidectomy, with or without insertion of a tympanostomy tube, in a family-centred and health literacy-focused manner in an Italian maternal and child health hospital compared to standard care. Moreover, objective of this study is also to evaluate the impact of the app on the surgical ward and other organisational aspects of the hospital compared to standard processes.

### Objectives {7}

Based on these premises, the following research questions will be addressed:

- Does an mHealth app designed to support caregivers of children undergoing tonsillectomy and/or adenoidectomy with or without tympanostomy tube insertion in the perioperative process improve family-health related outcomes compared to standard care?
- Does an mHealth app designed to support caregivers of children undergoing tonsillectomy and/or adenoidectomy with or without insertion of a tympanostomy tube improve organisational issues of the surgical ward in the perioperative process compared to standard care?

Therefore, the primary objective of the study is to determine the effectiveness of a plain-language mHealth app compared with other standard supportive and educational methods on anxiety of caregivers of children undergoing tonsillectomy and/or adenoidectomy with or without insertion of a tympanostomy tube.

Secondary objectives are to determine the effectiveness of a plain language mHealth app compared with other standard supportive and educational methods in terms of family preparation for hospitalisation and surgery, child distress, family satisfaction with care, and post-discharge management at home.

### Trial design {8}

A two-parallel arm, open randomised controlled trial is being conducted to evaluate the superiority of an mHealth app in reducing anxiety in caregivers of children undergoing tonsillectomy and/or adenoidectomy with or without insertion of a tympanostomy tube in the perioperative period compared to standard care. Standard care in the control group consists of information and education provided by nurses and physicians during preoperative visits and hospitalisation about the ORL perioperative process. The information and education are provided orally or through printed brochures. Eligible participants are randomly allocated in a 1:1 ratio to the intervention group (use of mHealth app) or the control group (standard care).

Our study protocol followed the SPIRIT guidelines [16].

## Methods: participants, interventions, and outcomes

### Study setting {9}

The study is monocentric and is being conducted in a 136-bed maternal and child health hospital in Northern Italy. In this hospital, a number of approximately 450 tonsillectomies or adeno-tonsillectomies are performed each year. Specifically, enrolment started December 16, 2022, and is taking place in the consulting rooms of the paediatric surgery ORL department. Data collection is being performed in the paediatric surgery ward and in the ORL department. Statistical data analysis will be held at the clinical epidemiology and public health research unit of the same hospital.

### Eligibility criteria {10}

The inclusion criteria are as follows: (1) caregivers of male and female children aged at least 2 years and no more than 10 years who are scheduled for tonsillectomy and/or adenoidectomy with or without insertion of a tympanostomy tube, (2) caregivers who are capable of oral and written Italian communication without impairment, and (3) caregivers who guarantee access to a smartphone and Internet connection. The exclusion criteria will be as follows: (1) caregivers with cognitive impairment,,(2) caregivers of children with cognitive impairment, (3) caregivers with visual impairment, (4) caregivers of children affected by chronic pain, (5) caregivers of children who had another surgical procedure in the previous month, and (6) caregivers who have never used at least one smartphone app.

### Who will take informed consent? {26a}

Nursing staff in the consultation rooms of the paediatric surgery ORL department are in charge of informing eligible caregivers about the study and obtaining signed informed consent from caregivers to voluntarily participate in the study.

### Additional consent provisions for collection and use of participant data and biological specimens {26b}

The present study does not involve the collection or derivation of data and biological specimen for purposes that are separate from the main trial.

## Interventions

### Explanation for the choice of comparators {6b}

Standard care was chosen as comparator. Standard care given to caregivers in the ORL perioperative process consists of information and education provided by nurses and physicians during preoperative visits and hospitalisation about the ORL perioperative process. Standard information and education include topics such as ORL surgical and anaesthesiologic techniques that will be performed or strategies for pain control at home after surgery. Information and education is usually provided orally and through printed brochures.

### Intervention description {11a}

The intervention group (group A) uses an a mHealth app for information and education to caregivers of children undergoing tonsillectomy and/or adenoidectomy with or without insertion of a tympanostomy tube in the perioperative process. Group A has the app in use from the day of enrolment to the day of follow-up visit. Group B receives standard care.

The content and functions of the mHealth app were developed in collaboration with an institute of maternal and child health and Area Science Park, both in Northeastern Italy. The methodological steps followed to develop the mHealth app were guided by the three cycles of the Information System Research Framework [17]. The mHealth app was developed using a user-centred participatory design approach that involved both caregivers of ORL surgical children (primary users) and hospital staff (secondary users) to understand their needs and expectations related to content topics and functionalities [18]. To promote an equally accessible understanding of the mHealth app content, a team of external communication experts was also consulted, and content, such as terminology, was simplified where necessary.

The information and education content provided in standard care to the control group by healthcare providers during hospital visits, either orally or in the form of brochures are similar to that provided to the intervention group through the medium of the mHealth app. Information topics included, for example, explaining of the surgical procedure chosen, the type of anaesthesia used, strategies for preparing children for hospitalisation and surgery, or how to manage pain at home after discharge. Because healthcare providers have little time available to inform the family about safe surgery and other topics due to the short hospital stay that characterises this type of surgery [8], the content in the app is expanded and caregivers have the opportunity to read it at the time that best suits them and with the time they need. In fact, all content is available to users at any time in a library section of the app. Moreover, in accordance with the principles of adult learning [19] and its application in telemedicine [20], the content of the application is adapted to the needs of the primary end-users and delivered “just in time” [20]. In particular, content is suggested to participants in the form of new content notifications that are available based on the specific perioperative period the child is in, such as the day of prehospital visits, the prehospital period, the day of hospitalisation and surgery, and the post-discharge period at home. Moreover, pop-up messages are displayed to the user as reminders of visits or documents to bring to the hospital. All the content is provided in text format and in Italian. This mHealth app has the potential to enable communication between primary and secondary end-users, but these features are not used by study participants and are not the subject of this study experiment.

The nurses from the ORL paediatric surgical department are in charge of caregivers of ORL surgical children enrolment. These nurses, who are familiar with all the content, functions and features of the mHealth app, are also responsible for instructing group A participants on (1) how to download the app to their smartphone or tablet and (2) how to use the mHealth app and what functions and content it offers. The app developers also created a three-minute presentation of the mHealth app, which is shown by the nurses on a tablet to the group A participants after enrolment. The mHealth app is available for group A from the day of the pre-surgery visit until the seventh day after surgery or follow-up, which usually totals 14 days. The iOS and Android app provided to group A complies with technical and sectoral regulations such as the GDPR and the NIS directive, meets standards such as ISO/IEC 27,001 and the CE label, and consists of the following: a web-based platform with a modular architecture for information support and dialogue with users, certified according to DM 93/42/CE in the class II A and meets the main sectoral requirements for functional and reliability. Moreover, the app platform and all related services are developed in accordance with the rules of the Three-Year Plan for Information Technology for Public Administration (2019/2021), as far as they are compatible, and in accordance to the “Cloud First” principle.

The app is available free of charge and is available to the hospital’s end-users. However, until the end of the trial, the app will only be available to group A study participants.

### Criteria for discontinuing or modifying allocated interventions {11b}

In the event of a child’s fever or other issues causing a delay in surgery, the possibility to use the app and its content in the correct time frames will be adjusted to reflect the new surgery date selected.

### Strategies to improve adherence to interventions {11c}

Considering the functionality of the app, for example, allowing content to be delivered “just in time” [20] and depending on the specific perioperative period and potential needs of the user, not only is caregiver education made more effective but also their interest in continuing to use the app is stimulated. In addition, pop-up notifications and reminders from the app assist group A participants in using the app and reading its content. Moreover, groups A and B participants are encouraged to attend scheduled visits where healthcare providers give ORL perioperative information and education.

### Relevant concomitant care permitted or prohibited during the trial {11d}

We consider and permit that participants of group A and B may be exposed during the study to information derived from consultation of other health care experts, books, or web and social media.

### Provisions for post-trial care {30}

If this study demonstrates evidence of the effectiveness of the mHealth app, the app will be available for free download and use by caregivers of children undergoing tonsillectomy and/or adenoidectomy at this hospital, with or without the insertion of a tympanostomy tube.

### Outcomes {12}

The primary outcome of the study is the difference between the intervention and control groups in terms of caregivers’ self-reported state anxiety. It is be measured using the State-Trait Anxiety Inventory form Y questionnaire [21] in the surgical department, before the caregiver and child go into the operating theatre on the day of surgery, approximately seven days after enrolment.

Secondary outcomes are:

- The difference between the intervention and control groups in caregiver preparation for hospitalisation and surgery, evaluated at arrival at the hospital using a checklist completed by the administrative nurse (e.g. number of documents missing on arrival) approximately 5 days after enrolment;
- The difference between the intervention and control groups in preparing children for surgery, evaluated by ORL surgical nurses on arrival at the surgical department, approximately 5 days after enrolment;
- The difference between the intervention and control groups in terms of children’s distress. It is assessed by ORL surgical nurses in the surgical department using the modified Yale Preoperative Anxiety Scale [22];
- The difference between the intervention and control groups in self-reported primary caregiver anxiety as measured by the State-Trait Anxiety Inventory form Y questionnaire [21] on the day of follow-up, around the seventh day after surgery and 13 days after enrolment.
- The difference between the intervention and control groups in terms of the social and health effects of the introduction of an mHealth app in a maternal and child hospital. It is evaluated on the day of follow-up, approximately 14 days after enrolment.

### Participant timeline {13}

Caregivers participate in the study from the day of enrolment to day 7 after surgery or follow-up. The assessment time points (T) for caregivers and children after enrolment and signing of the informed consent form are as follows:

- (T0) after enrolment and allocation, data collection at time zero—(a) demographic data and (b) caregiver anxiety trait and state; (c) caregiver health literacy. At T0, participants in group A receive the app (intervention), while group B continue to receive standard care;
- (T1) at hospital admission on the day of surgery—IRCCS administrative office (“Punto benvenuto”)—documents required for hospital admission and surgery;
- (T2) upon admission to the surgical department ORL—(a) caregiver’s state anxiety level; (b) child’s preparation for surgery; (c) child distress;
- (T3) at follow-up—caregiver’s state anxiety; social impact.

**Fig. 1:**
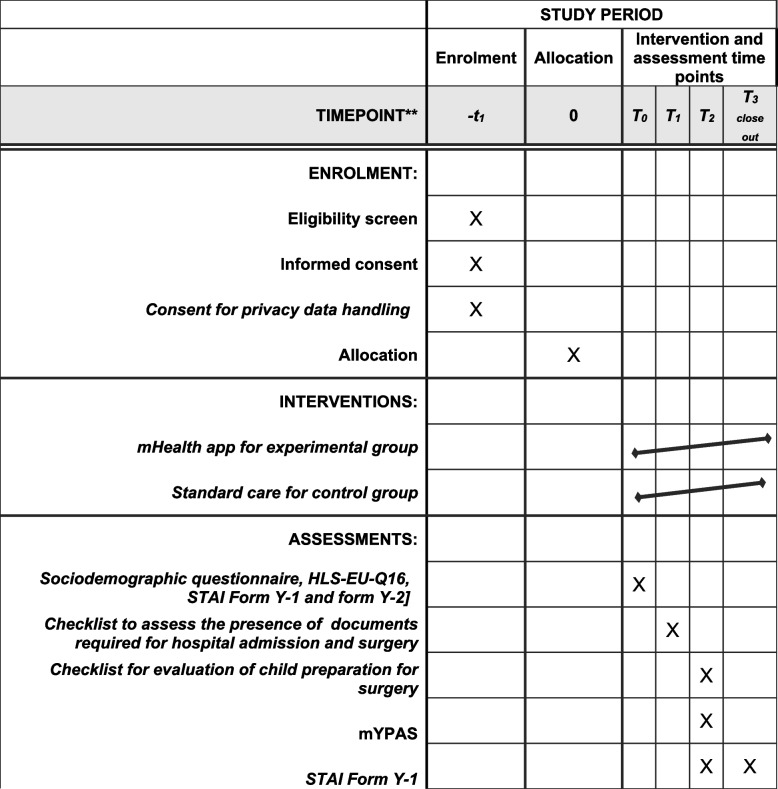
Timeline schedule of enrolment, interventions, and assessments

Participant timeline is shown schematically in Fig. 1.

### Sample size {14}

Assuming a mean score of preoperative anxiety (main outcome of the study) of 50 for the control group, with a standard deviation (SD) of 13 [21], and a mean of 45 for the intervention group, with SD of 10 (effect size = 0.43), an alpha value of 0.05 and a beta value of 0.20, 180 subjects (90 per group) are needed to conduct the study (G*power, Wilcoxon-Mann–Whitney test, two groups).

### Recruitment {15}

Caregivers of children selected for ORL surgery and accessing the hospital are screened according to inclusion and exclusion criteria. The caregivers are offered to participate in this study. Recruitment is expected to last 8 months and will continue until the estimated sample size is reached.

### Assignment of interventions: allocation

#### Sequence generation {16a}

Enrolled caregivers are randomly assigned into two groups (experimental group A and control group B) using a computerised number generation with simple randomisation and block randomisation with a block size of four to ensure a good balance of participants between the two groups.

#### Concealment mechanism {16b}

The allocation process is undertaken by the hospital’s Clinical Epidemiology and Public Health Research Unit to ensure random allocation and concealment. The allocation sequence is hidden from the researchers and other staff involved in the study in sealed, opaque, sequentially numbered envelopes.

#### Implementation {16c}

The nurse in the consultation rooms of the ORL paediatric surgery department is in charge to open the envelopes and give access to the app to group A, according to the number extracted and corresponding to the study arm.

### Assignment of interventions: blinding

#### Who will be blinded {17a}

Healthcare providers and participants could not be blinded in this study because the app in use is visible to caregivers and staff. Outcome assessors could not be blinded in this study.

#### Procedure for unblinding if needed {17b}

Because this study is an open-label study, no unblinding procedures are used.

## Data collection and management

### Plans for assessment and collection of outcomes {18a}

#### Assessment tools (groups A and B)

- A sociodemographic questionnaire is used to collect the following data at T0: age, sex, educational level of caregiver, current occupational status (employed, unemployed, retired, homemaker, student), relationship to child (mother, father, other guardian); being a healthcare professional; previous experience caring for others with health problems; age and sex of child; child’s planned surgery at this hospitalisation; child’s previous surgical experience.
- The level of health literacy is evaluated at T0 using the European Health Literacy Survey Questionnaire [23]. The HLS-EU-Q16 is a 16-item self-assessment tool with five possible answers on a Likert scale ranging from 1 (very difficult to) 4 (very easy), with the fifth possible answer being “I don’t know.” To determine the score, the possible answers HLS-EU-Q16 are dichotomised (‘don’t know’ answers are coded as missing values). ‘Fairly difficult’ and ‘very difficult’ are both coded 0 (zero), while ‘fairly easy’ and ‘very easy’ are both coded 1. HLS-EU-Q16 is a summated score with a range of 0–16. Based on the final score, three levels of health literacy (HL) can be defined: insufficient HL (0–8), problematic HL (9–12), sufficient HL (13–16) [23].
- The documents required for hospital admission and surgery is assessed at T1 using a checklist completed by administrative nurses (number of documents missing when the family arrives at the hospital, e.g. identity card, healthcare card).
- The child’s preparation for surgery is assessed at T2 by the nurse in the surgical department using a checklist that evaluates whether the child has adequate hygiene, is not wearing nail polish, is fasting, and is not wearing jewellery as prescribed by staff for standard surgical preparation.
- Caregivers’ anxiety is measured using the Italian version of the State-Trait Anxiety Inventory questionnaire [21]. The questionnaire consists of two self-report scales for measuring state anxiety and trait anxiety. The S-Anxiety scale (STAI Form Y-1) consists of twenty statements that evaluate how the respondent feels “now, at this moment,” on a 4 items Likert scale (from “not at all” to “very much”, attached). The T-Anxiety scale (STAI Form Y-2) consists of twenty statements that evaluate how the respondent feels “generally” on a four-point Likert scale, ranging from “almost never” to “almost always.” The total score ranges from 20 to 80 points, with lower scores indicating higher trait and state anxiety.

Caregivers are asked to complete both forms at T0 to determine anxiety trait and baseline levels. The state form of the STAI Form Y-1 is completed in the hospital surgical department prior to surgery at T2 and again on the seventh day after surgery (follow-up) at T3 to measure study outcomes**.**

- The modified Yale Preoperative Anxiety Scale (mYPAS) [22] is used as an observational tool to measure anxiety in children in the preoperative period at T2. The mYPAS consists of 27 items that examine five domains, such as child activity, emotional expressiveness, arousal state, vocalisation, and caregiver engagement. Scores range from 23.33 to 100, with higher scores indicating higher levels of anxiety. The tool is completed by nurses. Since there is no validated Italian version of the questionnaire, only English-speaking nurses are responsible for completing the instrument, according to Liguori and colleagues [24].
- The social and health impact of the study is self-assessed by caregivers at follow-up (T3) through outcome measures, such as how often caregivers had to call the hospital for additional information, had to visit the paediatric Emergency department, or had other complications at home after surgery (e.g. bleeding, uncontrolled pain).

The questionnaires at T0 and T2 are completed online via a tablet available to participants in groups A and B and to nurses in the surgical department. The assessment at T1 is completed in paper form. Moreover, considering that in this hospital not all caregivers bring their child for follow-up, sometimes preferring to see their general paediatrician, data collection at T3 is performed online after sending caregivers an email with a link to fill out the questionnaires.

Prior to the start of the study, nurses in the surgical department participating in the study were trained in the use of the assessment tools (e.g. mYPAS) with standardised instructions. The training was conducted by a nurse from the research ream in a meeting room of the department.

#### Other assessments (only group A)

- Data on app usage (number of logins; quality of content consulted, time spent on app) will be collected during experimentation of the app.
- At the end of the trial, participants are asked to provide an evaluation over the app’s content and features in digital form through the app itself.

### Plans to promote participant retention and complete follow-up {18b}

Data collection is conducted during the perioperative period when caregivers are in the hospital for visits and surgery, so participant presence in the hospital of participants eases data collection. As with any ORL surgical process at this hospital, participants in groups A and B are encouraged to attend the scheduled follow-up visit and are reminded by email to complete the questionnaires for the final assessments on T3, the day of follow-up.

### Data management {19}

Most data are collected electronically through an interface that complies with European (GDPR No. 679/2016) and Italian (D.L. 101/2018) data protection guidelines. Participants are pseudo-anonymised and given a ID number. Data collected at T1 on a paper form will be entered into an electronic record protected by a password. Two nurses will enter the data collected at T1 to verify correct entry. The data will be processed by the Clinical Epidemiology and Public Health Research Unit of the hospital using the SAS software, Version 9.4 (SAS Institute Inc., Cary, NC, USA) for data analysis. Access to the data will be restricted, and researchers and statisticians will be assigned a user ID and password.

### Confidentiality {27}

Confidentiality is ensured by assigning a unique participant code number to each participant to ensure pseudo-anonymisation. Confidentiality and anonymity of study participants are ensured according to European (GDPR No. 679/2016) and Italian (D.L. 101/2018) regulations.

### Plans for collection, laboratory evaluation, and storage of biological specimens for genetic or molecular analysis in this trial/future use {33}

No biological specimens will be collected.

## Statistical methods

### Statistical methods for primary and secondary outcomes {20a}

Description of the categorical variables will be made using frequency and percentage while mean and standard deviation (or median and interquartile range if variables are not normal) will be reported for continuous variables. Data will be analysed by intention-to-treat. A per-protocol analysis will be also performed. To evaluate the difference in the score of preoperative anxiety between intervention and control group, Wilcoxon-Mann–Whitney non-parametric test (or *t*-test, as necessary) will be applied. The same test will be calculated to evaluate the difference in the anxiety score between intervention and control group at T0 and T3.

Wilcoxon-Mann–Whitney will be also used to establish if a difference in the children distress will be between intervention and control group. Chi-square test or exact Fisher test will be applied to evaluate difference in the family preparation for hospitalisation and surgery at T1 between group A and B.

### Interim analyses {21b}

Interim analyses are not planned in this low-risk intervention consisting of providing information through a device.

### Methods for additional analyses (e.g. subgroup analyses) {20b}

No other additional or subgroup analyses are planned in this study.

### Methods in analysis to handle protocol non-adherence and any statistical methods to handle missing data {20c}

The main analysis is performed as “intention to treat.” All randomised subjects with an available main outcome will be analysed in the group to which they were randomised, regardless of whether they received the assigned intervention. Reasons for dropping out of the study are recorded in a data file and described in detail. A multiple imputation technique will be used for missing data.

### Plans to give access to the full protocol, participant-level data, and statistical code {31c}

The study protocol and data analysis will be available from the corresponding author on request.

## Oversight and monitoring

### Composition of the coordinating centre and trial steering committee {5d}

The IRCCS Burlo Garofolo is the coordinating centre and is responsible for providing day-to-day support to the surgical department staff and administrative nurses in organising the data collection and management of the study. The trial steering committee is composed of the study investigators and an epidemiologist from IRCCS Burlo Garofolo and a researcher from the Area Science Park. The members of the committee agreed on the final protocol and ensure that the study is conducted rigorously. They are responsible for all aspects of study management, from supervising participant enrolment to reviewing data quality. They have also trained the paediatric surgery nurses and administrative nurses in data collection. They monitor the overall conduct of the clinical trial and meet once a month to oversee the conduct and progress of the study.

### Composition of the data monitoring committee, its role and reporting structure {21a}

Considering the low risks of the study, no external data monitoring committee was appointed.

The trial steering committee monitor the conduct of the study and ensures that all phases of the trial are conducted with rigour.

### Adverse event reporting and harms {22}

The study aims to evaluate a low-risk intervention, and no serious adverse events are expected because the intervention consists of providing selected information through a device. Potential risks due incorrect use of the app were not considered relevant to mention.

### Frequency and plans for auditing trial conduct {23}

Considering the low-risk and brief intervention, no audits are planned for this study.

### Plans for communicating important protocol amendments to relevant parties (e.g. trial participants, ethical committees) {25}

If important protocol amendments are required, a request for approval of the changes will be forwarded to the regional ethics committee for approval. After approval, the new version of the protocol will be sent to ClinicalTrials.org. Finally, the entire research team will be informed of the changes at a meeting in a surgical department meeting room and through written information sent via institutional e-mail. Trial participants will be informed from the surgical ward nurses and study informed consent forms.

### Dissemination plans {31a}

The results of the study will be disseminated to the scientific community through scientific publications and presentations at conferences. In addition, the results of the study will be shared with citizens through the hospital’s website and through the media and social media.

## Discussion

Tonsillectomies and adenotonsillectomies are characterised by a short hospital stay and little time for healthcare providers to inform and educate families in a family-centred manner. To our knowledge, no mobile health application has been tested in Italy to support children, their caregivers, and, consequently, their healthcare professionals in the ORL perioperative period. Therefore, if the developed app proves successful, it could be useful for other Italian children undergoing these procedures and their families. Furthermore, it can be later translated into other languages to be available and helpful for other countries or foreigners in Italy. Such a solution can introduce a new and safe management model in paediatric healthcare. It provides families with access to an easily consumable format with evidence-based health content as part of the ORL operation process, leading to continuity of care and empowerment of citizens in terms of positive health management and access to health services.

In general, the app to be implemented aims to strengthen the offer of services to support collaboration between professionals and the interaction between professionals and citizens; to promote the empowerment of citizens in relation to health services; to propose and validate a new management model the health and social care.

The presented solution can enable a family-centred and easily consumable format of the presented evidence-based content and introduce a new management model in paediatric healthcare and education.

## Trial status

Study protocol RC 03/2022. Recruitment started on 16 December 2022. Time for enrolment since starting: 1 year*.* The estimated end date of the study is December 31, 2023.

## Data Availability

All data will be accessed by researchers of IRCSS Burlo Garofolo, University of Trieste and Area Science Park. Relevant data will be made accessible through publication to the scientific community and the public.

## Abbreviations

CE: Conformité Européene [in English: European Conformity]
D.L.: Decreto Legislativo [in English: Legislative Decree]
HLS-EU-Q16: European Health Literacy Survey Questionnaire
IEC: International Electrotechnical Commission
IRCCS: Istituti di Ricovero e Cura a Carattere Scientifico [in English: Scientific Hospitals and Care Institutes]
GDPR: General Data Protection Regulation
mHealth app: Mobile health application
NIS: Network and information systems
ORL: Otorhinolaryngology
SAS: Statistical analysis system
SD: Standard deviation
SPIRIT: Standard Protocol Items: Recommendations for Interventional Trials
mYPAS: Yale Preoperative Anxiety Scale
